# Identity-Based Provable Data Possession with Designated Verifier from Lattices for Cloud Computing

**DOI:** 10.3390/e27070753

**Published:** 2025-07-15

**Authors:** Mengdi Zhao, Huiyan Chen

**Affiliations:** Beijing Electronic Science and Technology Institute, Beijing 100070, China; 20232010@mail.besti.edu.cn

**Keywords:** provable data possession, leveled identity-based fully homomorphic signature, designated verifier, lattice

## Abstract

Provable data possession (PDP) is a technique that enables the verification of data integrity in cloud storage without the need to download the data. PDP schemes are generally categorized into public and private verification. Public verification allows third parties to assess the integrity of outsourced data, offering good openness and flexibility, but it may lead to privacy leakage and security risks. In contrast, private verification restricts the auditing capability to the data owner, providing better privacy protection but often resulting in higher verification costs and operational complexity due to limited local resources. Moreover, most existing PDP schemes are based on classical number-theoretic assumptions, making them vulnerable to quantum attacks. To address these challenges, this paper proposes an identity-based PDP with a designated verifier over lattices, utilizing a specially leveled identity-based fully homomorphic signature (IB-FHS) scheme. We provide a formal security proof of the proposed scheme under the small-integer solution (SIS) and learning with errors (LWE) within the random oracle model. Theoretical analysis confirms that the scheme achieves security guarantees while maintaining practical feasibility. Furthermore, simulation-based experiments show that for a 1 MB file and lattice dimension of *n* = 128, the computation times for core algorithms such as **TagGen**, **GenProof**, and **CheckProof** are approximately 20.76 s, 13.75 s, and 3.33 s, respectively. Compared to existing lattice-based PDP schemes, the proposed scheme introduces additional overhead due to the designated verifier mechanism; however, it achieves a well-balanced optimization among functionality, security, and efficiency.

## 1. Introduction

As information technology continues to advance rapidly, cloud computing has become a key component of modern information technology, particularly in the areas of data storage and processing, providing powerful support. As one of the core services of cloud computing, cloud storage enables users to store their local data on cloud servers, alleviating the storage burden on physical devices while enhancing the convenience and flexibility of data access [1,2]. However, as cloud storage becomes more widespread, outsourcing data to cloud service providers (CSPs) introduces security and integrity challenges.

One critical issue is ensuring the integrity of cloud-stored data. As cloud storage removes direct control over data, it exposes the data to potential attacks such as unauthorized tampering, malicious deletion, or even accidental damage due to hardware or software failures. Although users depend on CSPs to preserve data integrity, the cloud’s inherent openness and sharing raise significant issues regarding data security, privacy, and reliability. Whether due to malicious actions by CSPs or external attackers, there is a pressing need for robust mechanisms to ensure that outsourced data are properly stored and remain intact.

Real-world incidents further underscore this need. For example, during a six-month high-energy physics operation involving approximately 97 petabytes of data, CERN reported that about 128 megabytes had been irreversibly corrupted, raising concerns about the impact of even minor data degradation on scientific research outcomes [3]. In another case, Jeff Bonwick, creator of the ZFS file system, revealed that Greenplum—a high-performance database—experiences silent data corruption every 10 to 20 minutes without triggering system alerts. These cases illustrate that even in rigorously managed scientific and enterprise environments, data corruption can occur undetected, undermining both data reliability and decision making [3].

Traditional data integrity verification solutions, such as downloading the entire dataset for inspection, have become impractical due to the large data volumes involved and the high communication and computational costs [4]. To address this challenge, researchers have proposed various alternative approaches.

Among them, blockchain-based storage frameworks [5,6] leverage distributed ledgers and consensus protocols to achieve verifiability and tamper resistance. These schemes often utilize Merkle hash trees and smart contracts for auditability and state tracking. However, they typically suffer from high communication costs, significant computational overhead, and consensus-related delays, which limit their applicability in resource-constrained environments. On the other hand, coding techniques in edge computing [3,5] enhance data availability through redundant encoding and erasure coding, providing strong resilience against data loss or corruption. Nonetheless, these methods primarily focus on fault tolerance and recovery, and they lack fine-grained audit control or designated verifier mechanisms.

Intuitively, data owners can perform integrity verification tasks themselves, but this requires them to retrieve and check the data’s integrity individually, resulting in a significant communication and computational burden. To alleviate this burden, public auditing allows data users to delegate these tasks to a third-party auditor (TPA). Auditors can regularly check the integrity of outsourced data on behalf of the users. If the audit fails, the auditor quickly informs the user, indicating that the data may have been tampered with.

Although public auditing offers significant benefits, there are two main obstacles to its widespread application in cloud computing. On the one hand, many existing public auditing schemes [7,8,9,10,11] are based on traditional cryptographic hardness assumptions, which will be vulnerable to the emerging threats posed by quantum computing. With the inevitable rise of quantum computing, developing post-quantum secure auditing schemes becomes increasingly vital. On the other hand, current public auditing approaches depend on third-party auditors to verify data integrity. However, these schemes often demand significant computational resources from the auditors, as they involve intensive verification processes, including operations like bilinear pairings and modular exponentiations. These procedures place a heavy computational burden on auditors and create a performance bottleneck.

In addition to these challenges, the deployment of public auditing faces another security issue: public auditing may expose user privacy. Unauthorized third parties should not have access to sensitive user information. To protect data users’ privacy, users can designate a specific verifier to carry out data integrity checks.

In addition to existing security threats, the rapid development of quantum computing poses a fundamental challenge to data integrity verification mechanisms in cloud environments. Most widely adopted integrity verification methods are based on classical number-theoretic problems, such as integer factorization and discrete logarithms. However, these foundations are no longer secure in the face of quantum attacks—particularly because Shor’s algorithm [12] can efficiently solve these problems in polynomial time, rendering many cryptographic schemes that rely on them vulnerable. Once quantum computing becomes practical, existing integrity verification mechanisms will no longer provide long-term security guarantees, thereby seriously undermining the reliability and trustworthiness of cloud storage systems.

Consequently, designing data integrity verification mechanisms that are not only resistant to quantum attacks but also efficient in terms of computational, storage, communication, and transmission overhead has become a critical research direction for securing the next generation of cloud storage infrastructures.

### 1.1. Related Work

PDP is a practical method for verifying the outsourced data’s integrity, and it was first introduced by Ateniese et al. [13] based on the integer factorization hypothesis. PDP generates verifiable metadata during the data processing phase, which are then outsourced to the cloud service provider (CSP). Afterward, the verifier checks the data’s integrity by randomly sampling blocks. For instance, Ateniese and colleagues demonstrated that with a file containing 10,000 blocks, the verifier can achieve a 99% error detection rate by requesting proofs from just 460 randomly selected blocks [14].

Subsequently, a variety of PDP mechanisms have been developed to suit the diverse requirements of various cloud deployment environments, such as dynamic scenarios, batch verification, and privacy protection. For instance, Yuan et al. [15] developed a new dynamic PDP scheme with multiple replicas to verify the integrity of files stored by users across multiple CSPs. He et al. [7] introduced a PDP scheme for shared data that enables completely dynamic updates and ensures that verifier storage costs remain constant. Zhang et al. [16] tackled enterprise private cloud data sharing challenges by employing attribute-based signatures to design a revocable integrity auditing scheme under the SIS problem. Focusing on the key management issue, Zhang et al. [17] developed an identity-based public auditing scheme with key-exposure resistance based on lattices, which updates the user’s private key using lattice basis delegation while maintaining a constant key size. Wang et al. [18] designed an identity-based data integrity auditing scheme from the lattice method, ensuring forward security. In short, identity-based PDP schemes can simplify key management, thereby reducing the burden on both CSPs and data owners. Sasikala and Bindu [19] introduced a certificateless batch verification scheme over lattices designed to support integrity checking of multiple cloud-stored files. To address the inherent private key escrow problem and lower the overhead of managing public key certificates, Zhou et al. [8] developed a certificate-based PDP scheme under the square computational Diffie–Hellman (CDH) assumption. Zhang et al. [20] developed a revocable certificateless PDP scheme that preserves user identity privacy while also eliminating the key escrow and certificate management problems found in traditional approaches, supporting efficient user revocation.

The abovementioned schemes, referred to as public PDP schemes [21], allow anyone to verify data integrity without downloading the full data. In these schemes, the proofs produced by cloud servers can be validated by anyone.

In contrast to public PDP schemes, Shen and Tzeng [22] introduced a delegatable PDP scheme in 2011, enabling the data owner to create a delegation key for the designated verifier, which is then stored on the cloud server for later verification. The following year, Wang [23] introduced the concept of proxy PDP and provided a concrete construction, allowing users to delegate auditing authority to a proxy through a delegation warrant. In fact, both of the previously discussed methods fall under the category of PDP with designated verifier (DV-PDP) schemes. In the DV-PDP scheme, the user can designate a specific verifier (proxy) to conduct the outsourced data’s integrity verification on their behalf. However, both DV-PDP schemes [22,23] were demonstrated to be insecure by Ren et al. [24], as a dishonest cloud storage server could obtain the key information associated with the delegated verifier. Unfortunately, Zhang et al. [25] demonstrated that Ren et al.’s [24] scheme is insecure and vulnerable to forgery attacks. In 2017, Wu et al. [21] introduced the earliest non-repudiable DV-PDP scheme aimed at addressing the non-repudiation issue and reducing possible conflicts between users and CSPs. Zhang et al. [26] proposed a lattice-based designated verifier auditing scheme specifically designed for cloud-assisted wireless body area networks, which ensures that only the designated verifier is capable of verifying the integrity of outsourced medical data stored on the associated cloud server. To address the vulnerability of DV-PDP to replay attacks launched by malicious cloud servers, a remote data possession verification scheme with a designated verifier was proposed by Yan et al. [27] under the CDH assumption, ensuring that only the specified verifier can validate data integrity, while others are unable to do so. However, this approach depends on public key infrastructure technology and fails to tackle data privacy concerns. To address these limitations, Bian et al. [28] designed an identity-based remote data possession verification scheme based on the discrete logarithm and CDH assumptions, allowing data owners to designate a specific verifier.

### 1.2. Contribution

To address the challenge of constructing a post-quantum PDP scheme that supports both identity-based cryptosystem and designated verifier auditing, this paper proposes a lattice-based PDP framework tailored for secure and accountable cloud storage in the post-quantum era. The key contributions of this paper are summarized as follows:

- This paper proposes a novel identity-based PDP scheme that employs a specially leveled IB-FHS scheme to eliminate the complexity of traditional public key infrastructures, thereby simplifying key management.
- The proposed scheme introduces a designated verifier mechanism, ensuring that only authorized auditors can perform legitimate data integrity checks. This effectively mitigates the privacy risks associated with public verifiability and enhances the controllability and accountability of the auditing process.
- The scheme is proven secure under the SIS and LWE assumptions in the random oracle model, ensuring its resistance against quantum attacks.
- This paper conducts a comprehensive evaluation of the proposed scheme through theoretical analysis and simulation-based experiments, covering communication overhead, storage requirements, and computational cost. The experimental results under representative parameter settings demonstrate that the core algorithms maintain reasonable computation times. Compared to existing PDP schemes, although the introduction of a designated verifier mechanism leads to a certain increase in computational overhead, the proposed scheme achieves a well-balanced tradeoff between functionality and efficiency.

Overall, our work aims to construct an identity-based PDP with designated verifier scheme over lattices, offering quantum-resistant security and flexible auditing control. It is particularly suitable for cloud auditing scenarios that require authorization verification in a post-quantum setting.

### 1.3. Organization

The rest of this paper is organized as follows. Section 2 introduces the necessary preliminaries. Section 3 defines our PDP scheme and its security model. Section 4 presents the detailed construction of the proposed PDP scheme. Section 5 provides formal security analysis covering unforgeability, indistinguishability, and robustness. Section 6 offers a performance evaluation in terms of computation, storage, and communication. Finally, Section 7 provides the conclusion.

## 2. Preliminaries

### 2.1. Notation

In this paper, we apply some initial symbols, as shown in Table 1.

### 2.2. Lattice

A lattice Λ in $R^{m}$ is a discrete additive subgroup generated by integer linear combinations of basis vectors. Given a matrix $A=[a_{1}\left|\cdots \right|a_{n}]\in R^{m\times n}$ with column vectors $a_{i}$, the lattice is defined as

(1) $$\Lambda =\left\{\sum_{i=1}^{n}x_{i}a_{i}|x_{i}\in Z\right\}\subseteq R^{m}.$$

The value *n* is called the rank of Λ. If n=m, then Λ is said to be full rank.

The dual lattice $\Lambda^{*}$ consists of all vectors $y\in R^{m}$ such that 〈x,y〉∈Z for all x∈Λ. When A is a basis of Λ, the basis of $\Lambda^{*}$ can be written as $B^{*}=B{\left(B^{T}B\right)}^{-1}$.

In this paper, we focus on *q*-ary lattices, i.e., full-rank lattices that contain $qZ^{m}$.

**Definition** **1** (*q*-ary lattice)**.** *Given $B\in Z^{n\times m},k\in Z_{q}^{n}$, the following definitions describe q-ary lattices:*

(2) $$\begin{array}{cc} \Lambda_{q}\left(B\right) & :=\{a\in Z^{m}\;|\;\exists e\in Z_{q}^{n}\;\mathrm{such}\;\mathrm{that}\;B^{T}e=a\;\mathrm{mod}q\},\\ \Lambda_{q}^{⊥}\left(B\right) & :=\{a\in Z^{m}\;|\;\mathrm{Ba}=0\;\mathrm{mod}q\},\\ \Lambda_{q}^{k}\left(B\right) & :=\{a\in Z^{m}\;|\;\mathrm{Ba}=k\;\mathrm{mod}q\}, \end{array}$$

*where if $y\in \Lambda_{q}^{k}\left(B\right)$, then $\Lambda_{q}^{k}\left(B\right)=\Lambda_{q}^{⊥}\left(B\right)+y$.*

**Definition** **2** (Gaussian distribution on lattice)**.** *Let $u\in R^{m}$ and φ∈R define the following: $\rho_{\varphi ,u}\left(t\right)=\exp\left(\pi \frac{{∥t-u∥}^{2}}{\varphi^{2}}\right)$ and $\rho_{\varphi ,u}\left(\Lambda \right)=\sum_{t\in \Lambda }\rho_{\varphi ,u}\left(t\right)$. The discrete Gaussian distribution on lattice* Λ, *centered at u with parameter φ, is then given by $\forall y\in \Lambda ,D_{\Lambda ,\varphi ,u}\left(y\right)=\frac{\rho_{\varphi ,u}\left(y\right)}{\rho_{\varphi ,u}\left(\Lambda \right)}$.*

**Lemma** **1** (Leftover hash lemma [29])**.** *Let q>2,m>(n+1)logq+ω(logn), $Q\in {\left\{-1,1\right\}}^{m\times k}$, $J\in Z_{q}^{n\times m},B\in Z_{q}^{n\times k}$ be randomly selected matrices, where k=k(n) is a polynomial. The distributions $\left(J,\mathrm{JQ},Q^{T}d\right)$ and $\left(J,B,Q^{T}d\right)$ are statistically indistinguishable for any vector $d\in Z^{m}$.*

### 2.3. Trapdoor and Sample Algorithms from Lattices

We now present the algorithms used for trapdoor generation and lattice-based sampling. In our construction, the public key and master secret key are generated using the efficient randomized algorithm TrapGen. To derive private keys for the data owner and the designated verifier, we employ the SampleBasisLeft and NewBasisDel algorithms, respectively. For generating tags on data blocks, we utilize the SampleLeft algorithm.

**Lemma** **2** ($\mathrm{TrapGen}(1^{n},1^{m},q)$ [29])**.** *Given n≥1,q≥2,m=O(nlogq), we can obtain $\left(U,P\right)\leftarrow \mathrm{TrapGen}\left(1^{n},1^{m},q\right)$, where the distribution of $U\in Z_{q}^{n\times m}$ is statistically close (within negligible distance in n) to uniform over $Z_{q}^{n\times m}$, and P is U’s trapdoor.*

**Definition** **3** (Gadget matrix)**.** *Given m=n·⌈logq⌉, the gadget matrix G is defined as $G=g⊗I_{n}\in Z_{q}^{n\times m}$, where $g=\left(1,2,4,\cdots ,2^{\left\lceil \log q\right\rceil }\right)\in Z_{q}^{\left\lceil \log q\right\rceil }$. The inverse function $G^{-1}:Z_{q}^{n\times m}\to {\left\{0,1\right\}}^{m\times m}$ converts each entry $x\in Z_{q}$ of matrix A into a column of its binary representation. For any $A\in Z_{q}^{n\times m}$, $G_{n}\cdot G_{n}^{-1}\left(A\right)=A$.*

**Lemma** **3** (SampleLeft(J,Q,P,k,s) [30])**.** *Let us have $q\geq 2,m\geq n,s\geq ∥\tilde{P}∥\cdot \omega \left(\sqrt{\log(m+m_{1})}\right)$, matrices $J\in Z_{q}^{n\times m},Q\in Z^{n\times m_{1}}$, J’s trapdoor P, and vector $k\in Z_{q}^{n}$; then, we can get $e\in Z^{m+m_{1}}\leftarrow \mathrm{SampleLeft}\left(J,Q,P,k,s\right)$, where e is distributed statistically close to $D_{\Lambda_{q}^{k}\left(J|Q\right),s}$. Accordingly, with the delegated algorithm SampleBasisLeft(J,Q,P,s), it is possible to output $\Lambda_{q}^{⊥}\left(J|Q\right)$’s basis as $P_{\left(J|Q\right)}$.*

**Lemma** **4** ($\mathrm{SampleRight}(J,Q,H,P_{Q},k,s)$ [30])**.** *For given q>2,m>n, $J\in Z_{q}^{n\times k},Q\in Z^{n\times m},H\in Z^{k\times m}$, Q’s trapdoor $P_{Q}$ and $k\in Z_{q}^{n}$, we can get $c\in Z^{m+k}\leftarrow \mathrm{SampleRight}(J,Q,H,$$P_{Q},k,s)$, where c is statistically close to the distribution $D_{\Lambda_{q}^{k}\left(J|\mathrm{JH}+Q\right),s}$, $s>∥{\tilde{P}}_{Q}∥\cdot s_{H}\cdot \omega \left(\sqrt{\log m}\right)$ and to $s_{H}=\sup_{∥y∥=1}∥Hy∥$. Furthermore, with the delegated algorithm SampleBasisRight $\left(J,Q,H,P_{Q},s\right)$, it is possible to output $\Lambda_{q}^{⊥}\left(J|\mathrm{JH}+Q\right)$’s basis as $P_{\left(J|\mathrm{JH}+Q\right)}$.*

**Lemma** **5** (NewBasisDel(J,H,P,s) [31])**.** *Given q>2,m>2nlogq, $J\in Z_{q}^{n\times m},H\in D_{m\times m}$, and J’s trapdoor P, we get $P_{Q}\leftarrow \mathrm{NewBasisDel}\left(J,H,P,s\right)$, where $P_{Q}$ is $\Lambda_{q}^{⊥}\left(Q={\mathrm{JH}}^{-1}\right)$’s basis, $s>∥\tilde{P}∥\cdot s_{H}\sqrt{m}\cdot \omega \left(\log^{3/2}m\right),s_{H}=\sqrt{n\log q}\cdot \omega \left(\sqrt{\log m}\right)$, and $D_{m\times m}$ follows the discrete Gaussian distribution $D_{Z^{m},s_{H}}$.*

**Lemma** **6** (SampleRwithBasis(J) [30])**.** *Given $q>2,m>2n\log q,J\in Z_{q}^{n\times m}$, SampleRwithBasis(J) produces an invertible matrix $H\in D_{m\times m}$ and $\Lambda_{q}^{⊥}\left(Q={\mathrm{JH}}^{-1}\right)$’s basis $P_{Q}$, where $∥{\tilde{P}}_{Q}∥\leq O\left(\sqrt{n\log q}\right)$.*

**Lemma** **7** (SampleD(J,P,k,s) [29])**.** *Given $J\in Z_{q}^{n\times m},k\in Z_{q}^{n}$, $\Lambda_{q}^{⊥}\left(J\right)$’s basis P, and $s=O(\sqrt{n\log q})$, SampleD(J,P,k,s) samples $v\in Z_{q}^{n}$ from the discrete Gaussian distribution $D_{\Lambda_{q}^{k}\left(J\right),s\cdot \omega \left(\sqrt{\log n}\right)}$.*

### 2.4. Hard Problems on Lattices

**Definition** **4** (${\mathrm{SIS}}_{n,m,q,\beta }$ problem)**.** *Given n,m,q,β and $q\geq \beta \cdot (\sqrt{n\log n})$, the ${\mathrm{SIS}}_{n,m,q,\beta }$ problem is to find a vector $k\in Z_{q}^{n}\setminus \left\{0\right\}$ with ∥k∥≤β such that Bk=0, where $B\in Z_{q}^{n\times m}$ is a uniform and random matrix. The average case ${\mathrm{SIS}}_{n,m,q,\beta }$ problem is as difficult as solving the worst case ${\mathrm{GapSVP}}_{\tilde{O}\left(\beta \cdot \sqrt{n}\right)}$ [29].*

**Definition** **5** (${\mathrm{LWE}}_{n,m,q,\beta }$ problem)**.** *Given n,m≥n,q≥2 and $\beta q>2\sqrt{n}$, then randomly select vectors $s\in Z_{q}^{n},k\in Z_{q}^{m}$ and a uniform matrix $B\in Z_{q}^{n\times m}$, as well as sample a Gaussian vector $r\leftarrow {\left(D_{Z^{m},\sqrt{2}\beta q}\right)}^{m}$, and the distributions $\left(B,B^{T}s+r\right)$ and (B,k) are indistinguishable. The ${\mathrm{LWE}}_{n,m,q,\beta }$ problem is as hard as SIVP under quantum reductions to within O(n/β) [30].*

For the LWE instance $\left(B,B^{T}s+r\right)$, if the Euclidean norm of B’ trapdoor P is sufficiently small and if r follows a discrete Gaussian distribution, then s can be easily retrieved, as explained in [32]. It is important to note that $P^{T}\left(B^{T}s+r\right)\left(\mathrm{mod}q\right)={\left(\mathrm{BP}\right)}^{T}s+P^{T}r\left(\mathrm{mod}q\right)$, where P and r both contain very small entries, and $P^{T}r\left(\mathrm{mod}q\right)=P^{T}r$. Then, multiply by ${\left(P^{T}\right)}^{-1}$ to obtain r. Moreover, we can easily recover s.

### 2.5. Pseudo-Random Functions

For any ℓ≥1, a pseudo-random function (PRF) is defined by a pair of the probabilistic polynomial-time (PPT) algorithms PRF=(PRF.Gen,PRF.Eval) as follows:

- $\mathrm{PRF}.\mathrm{Gen}(1^{\lambda })$: Output a seed $\varphi_{1}\in Z_{q}^{*}$ under the security parameter λ.
- $\mathrm{PRF}.\mathrm{Eval}(\varphi_{1},i)$: Given $\varphi_{1}\in Z_{q}^{*}$ and i∈[ℓ], return $\zeta_{i}\in Z_{q}$. For convenience, we use ${\mathrm{PRF}}_{\varphi_{1}}\left(\cdot \right)$ to represent the algorithm PRF.Eval, where ${\mathrm{PRF}}_{\varphi_{1}}\left(\cdot \right):Z_{q}^{*}\times \left\{1,2,\cdots ,\ell \right\}\to \left\{\zeta_{1},\cdots ,\zeta_{\ell }\right\}$.

### 2.6. Pseudo-Random Permutations

For any ℓ≥1, a pseudo-random permutation (PRP) is defined by a pair of PPT algorithms PRP=(PRP.Gen,PRP.Eval) as follows:

- $\mathrm{PRP}.\mathrm{Gen}(1^{\lambda })$: Output a seed $\varphi_{2}\in Z_{q}^{*}$ under the security parameter λ.
- $\mathrm{PRP}.\mathrm{Eval}(\varphi_{2},i)$: Given $\varphi_{2}\in Z_{q}^{*}$ and i∈[ℓ], return $\psi_{i}\in \left[\ell \right]$. For convenience, we use ${\mathrm{PRP}}_{\varphi_{2}}\left(\cdot \right)$ to represent the algorithm PRP.Eval, where ${\mathrm{PRP}}_{\varphi_{2}}\left(\cdot \right):Z_{q}^{*}\times \left\{1,2,\cdots ,\ell \right\}\to \left\{\psi_{1},\cdots ,\psi_{\ell }\right\}.$

It should be noted that the elements in the set $\left\{\psi_{1},\cdots ,\psi_{\ell }\right\}$ are distinct.

## 3. Framework of Our Provable Data Possession

### 3.1. System Model

**Figure 1:**
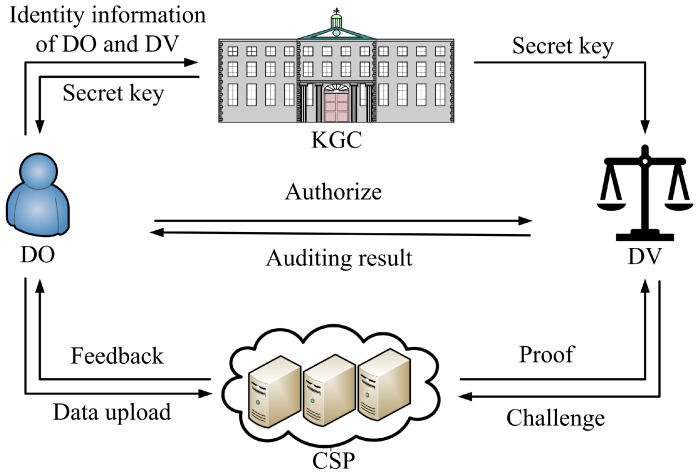
System model.

The system model of our PDP scheme involves four primary entities: the key generation center (KGC), the data owner (DO), the CSP, and the designated verifier (DV). The PDP scheme comprises four entities, as depicted in Figure 1. Their roles and responsibilities are described as follows:

- KGC: The KGC is a globally trusted authority in the system. It is tasked with generating the public parameter and master secret key. In addition, the KGC produces the data owner and the designated verifier’s private keys based on their identities.
- DO: To reduce storage and management burdens, the data owner outsources their data to CSPs. Moreover, the data owner can specify a particular verifier who is exclusively authorized to perform integrity checks on the outsourced data.
- CSP: With ample computational resources and storage space, the CSP provides data storage services to users. However, it is untrusted, meaning it may delete or tamper with the outsourced data for its own benefit or deceive the user for reputation.
- DV: The designated verifier is a trusted entity explicitly appointed by the data owner to conduct data integrity verification. Unlike publicly verifiable schemes where any party can audit the data, the designated verifier model restricts the ability to verify to a specific, authorized party.

### 3.2. Syntax

The proposed PDP scheme Π includes six algorithms: Π= (**Setup**, **KeyGenS**, **KeyGenV**, **TagGen**, **GenProof**, and **CheckProof**). The following PPT algorithms constitute our PDP scheme:

1. $\mathrm{Setup}(1^{\lambda },1^{l})$: Given security parameter λ and the maximum data blocks *l*, produce public parameter params and master secret key msk. For simplicity, params is implicitly treated as an input for the remaining algorithms.
2. $\mathrm{KeyGenS}(msk,id_{A})$: Upon the input of master secret key msk and the identity $id_{A}$, produce the public/private key $\left(PK_{A},SK_{A}\right)$ of the data owner.
3. $\mathrm{KeyGenV}(msk,id_{B})$: Upon the input of master secret key msk and the identity $id_{B}$, produce the public/private key $\left(PK_{B},SK_{B}\right)$ of the designated verifier.
4. $\mathrm{TagGen}(PK_{A},SK_{A},PK_{B},U)$: Given public keys $PK_{A},PK_{B}$, secret key $SK_{A}$, and file U, the data owner splits U into *l* blocks (with zero padding if needed), i.e., $U:=(U_{1},\cdots ,U_{l})$, where $U_{i}\in Z_{q}^{n\times n}$ for i∈[l]; return $\Omega^{id}={\left\{\left(U_{i},u_{i}\right)\right\}}_{i\in [l]}$, where $\left(U_{i},u_{i}\right)$ represents the *i*-th block–tag pair. Let $\Omega^{id}$ be the set of all block–tag pairs.
5. $\mathrm{GenProof}(PK_{A},PK_{B},\Omega^{id},CH)$: Given public keys $PK_{A},PK_{B}$, block–tag pairs $\Omega^{id}$, and the designated verifier’s challenge CH, return a PDP proof $\Sigma^{id}$.
6. $\mathrm{CheckProof}(PK_{A},PK_{B},SK_{B},CH,\Sigma^{id})$: Given public keys $PK_{A},PK_{B}$, secret key $SK_{B}$, the challenge CH, and the PDP proof $\Sigma^{id}$, output 1 (accept) or 0 (reject).

**Figure 2:**
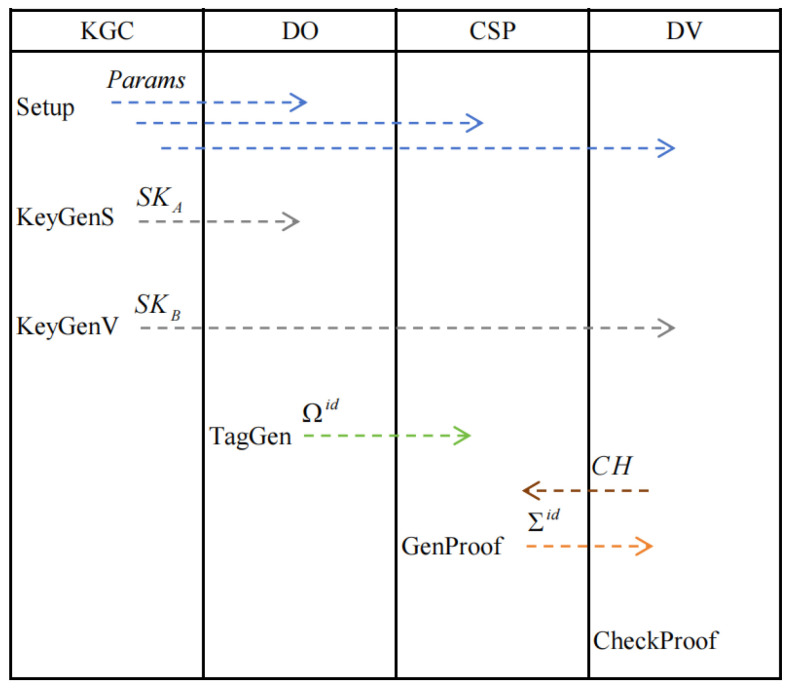
Algorithm steps.

In Figure 2, the algorithm shown below the entity name indicates that the entity is responsible for executing the algorithm. The dashed arrow indicates that the algorithm output is transmitted to the designated receiver.

**Correctness:** Given $\left(params,msk\right)\leftarrow \mathrm{Setup}\left(1^{\lambda },1^{l}\right)$, for all $msk,id_{A},id_{B},U,CH$, $\left(PK_{A},SK_{A}\right)\leftarrow \mathrm{KeyGenS}\left(msk,id_{A}\right)$, $\left(PK_{B},SK_{B}\right)\leftarrow \mathrm{KeyGenV}\left(msk,id_{B}\right)$, and $\Omega^{id}\leftarrow \mathrm{TagGen}\left(PK_{A},SK_{A},PK_{B},U\right)$, if $\Sigma^{id}\leftarrow \mathrm{GenProof}\left(PK_{A},PK_{B},\Omega^{id},CH\right)$, then $\mathrm{CheckProof}(PK_{A},PK_{B},SK_{B},CH,\Sigma^{id})=1$.

### 3.3. Security

The unforgeability proof shows that a malicious CPS, acting as an adversary, cannot deceive the designated verifier or pass the verification by submitting a forged response auditing proof.

For the scheme Π, the selectively unforgeable proof is defined by the game ${\mathrm{Exp}}_{A}\left(1^{\lambda }\right)$ between the PPT adversary A and the challenger C. Let $id_{A},id_{B}$ denote identities of Alice and Bob, respectively. The game is defined as follows:

- **Initial:** A announces to C the target identities $id_{A^{*}}$ and $id_{B^{*}}$, as well as a list of γ messages $U_{1},\cdots ,U_{\gamma }$ under the target identity $id_{A^{*}}$ denoted as $U:=\{U_{1},\cdots ,U_{\gamma }\}$ for 0≤γ≤l.
- **Setup:** C gets $\left(params,msk\right)\leftarrow \mathrm{Setup}\left(1^{\lambda },1^{l}\right)$ and provides params to A while keeping msk confidential.
- **Key queries:** A queries any identity $id_{A},id_{B}$’s secret key (except $id_{A^{*}},id_{B^{*}}$). C gets $\left(PK_{A},SK_{A}\right)$ using $\mathrm{KeyGenS}(msk,id_{A})$ and $\left(PK_{B},SK_{B}\right)$ using $\mathrm{KeyGenV}(msk,id_{B})$ and then transmits them to A.
- **Block–tag queries:** A sends U under the identity $id_{A^{*}}$ to C. C returns block–tag pairs $\Omega^{id_{A^{*}}}={\left\{\left(U_{i},u_{i}^{id_{A^{*}}}\right)\right\}}_{i\in [\gamma ]}\leftarrow \mathrm{TagGen}\left(PK_{A},SK_{A},PK_{B},U\right)$ to A.
- **PDP proof queries:** A transmits a challenge CH limited to U. Using $\Omega^{id_{A^{*}}}$, C produces a PDP proof $\Sigma^{id_{A^{*}}}\leftarrow \mathrm{GenProof}\left(PK_{A},PK_{B},\Omega^{id_{A^{*}}},CH\right)$ and transmits it to A.
- **Challenge:** C produces $CH^{id_{A^{*}}}$ restricted to U and transmits it to A.
- **Forgery:** A generates a forgery ${\hat{\Omega }}^{id_{A^{*}}}$.

A wins the game if either condition (1) or (2) is satisfied. These conditions are defined as follows:

(1) The PDP proof ${\hat{\Omega }}^{id_{A^{*}}}$ passes the verifier’s check.

(2) The PDP proof meets ${\hat{\Omega }}^{id_{A^{*}}}\neq \Omega^{id}$, where $\Omega^{id}$ is an honest PDP proof.

Let ${\mathrm{Adv}}_{A}$ represent the advantage of A in winning the experiment based on the coin tosses of A and C. This defines the security for our PDP scheme.

**Definition** **6** (Unforgeability)**.** *For given the security parameters λ and the maximum number of data blocks l, if no PPT adversary A can succeed in the game ${\mathrm{Exp}}_{A}\left(1^{\lambda }\right)$ with non-negligible probability, i.e., ${\mathrm{Adv}}_{A}$ is negligible, then the proposed scheme is considered selectively unforgeable.*

## 4. Our Provable Data Possession Scheme

In this section, we propose an identity-based PDP with a designated verifier, which is constructed using leveled IB-FHS. Building upon the approach in [14], the proposed scheme introduces the designated verifier mechanism that ensures that only the designated verifier can check for data integrity. Specifically, we pre-encode the messages to be signed into matrix form, use the leveled IB-FHS in combination with the designated verifier’s public key information to produce signatures, and treat these signatures as tags for the file, which are then verified by the designated verifier. The lattice-based PDP scheme is constructed as follows.

Let $id_{A}$ and $id_{B}$ represent the identities of data owner Alice and designated verifier Bob, respectively. The hash function refers to the full-rank difference (FRD) map $H_{1}:Z_{q}^{n}\to Z_{q}^{n\times n}$ [29]. $H_{2}:{\left\{0,1\right\}}^{*}\to D_{m\times m}$ and $H_{3}:{\left\{0,1\right\}}^{*}\times Z_{q}^{n\times n}\to Z_{q}^{2m\times m}$ are collision-resistant hash functions.

### 4.1. Construction

1. $\mathrm{Setup}(1^{\lambda },1^{l})$ is shown in Algorithm 1. Given the security parameter λ and the maximum number of data blocks *l*, return the public parameters params and the master secret key msk.

**Algorithm 1: Setup** $(1^{\lambda },1^{l})$
**    Ensure:** 
params and msk      1:Set $n=n\left(\lambda ,l\right),q=q\left(n,l\right),m_{0}=n\left(n,l\right)$, $m_{1}=n\left\lceil \log q\right\rceil$ and $m=m_{0}+m_{1}$      2:Pick Gaussian parameters $s_{1},s_{2},s_{3}$      3:

$\left(A_{0},T_{A_{0}}\right)\leftarrow \mathrm{TrapGen}\left(1^{n},1^{m_{0}},q\right)$

      4:Sample random matrices $A_{1},B,{\left\{D_{i}\right\}}_{i\in [l]}\in Z_{q}^{n\times m_{1}}$ and $A_{2}\in Z_{q}^{n\times m}$      5:**Return** $params=(A_{0},A_{1},A_{2},B,{\left\{D_{i}\right\}}_{i\in [l]})$ and $msk=T_{A_{0}}$

2. $\mathrm{KeyGenS}(msk,id_{A})$ is shown in Algorithm 2. Given msk and the identity $id_{A}$, return the public key and secret key of Alice.

**Algorithm 2:**

$\mathrm{KeyGenS}(msk,id_{A})$


**    Ensure:** 


$\left(PK_{A},SK_{A}\right)$

      1:Compute $F_{id_{A}}=A_{1}+H_{1}\left(id_{A}\right)B\in Z_{q}^{n\times m_{1}}$      2:Set identity matrix $A_{id}=\left[A_{0}|F_{id_{A}}\right]\in Z_{q}^{n\times m}$      3:Run $\mathrm{SampleBasisLeft}(A_{0},F_{id_{A}},T_{A_{0}},s_{1})$ to obtain $T_{A_{id}}$, which is a basis of $\Lambda_{q}^{⊥}\left(A_{0}|F_{id_{A}}\right)$, where $s_{1}\geq ∥{\tilde{T}}_{A_{0}}∥\cdot \omega \left(\sqrt{\log m}\right)$ according to Lemma 3      4:**Return** Alice’s public/secret key $\left(PK_{A},SK_{A}\right)=\left(A_{id},T_{A_{id}}\right)$

3. $\mathrm{KeyGenV}(msk,id_{A},id_{B})$: Given msk and the identities $id_{A},id_{B}$, then run Algorithm 3 to return the public key and secret key of Bob.

**Algorithm 3:** $\mathrm{KeyGenV}(msk,id_{A},id_{B})$
**    Ensure:** 


$\left(PK_{B},SK_{B}\right)$

      1:Compute $F_{id_{B}}=H_{2}\left(id_{B}\right)\in D_{m\times m}$ and $B_{id}=A_{id}F_{id_{B}}^{-1}\in Z_{q}^{n\times m}$      2:Run $\mathrm{NewBasisDel}(A_{id},F_{id_{B}},T_{A_{id}},s_{2})$ to get $T_{B_{id}}$, where $s_{2}>∥{\tilde{T}}_{A_{id}}∥\cdot s_{H}\sqrt{m}\cdot \omega \left(\log^{3/2}m\right)$ and $s_{H}=\sqrt{n\log q}\cdot \omega \left(\sqrt{\log m}\right)$ according to Lemma 6      3:**Return** Bob’s public/secret key $\left(PK_{B},SK_{B}\right)=\left(B_{id},T_{B_{id}}\right)$

4. $\mathrm{TagGen}(PK_{A},SK_{A},PK_{B},U)$ is shown in Algorithm 4. Upon inputting public keys $PK_{A},PK_{B}$, secret key $SK_{A}$, and a file U, Alice outputs $\left(U,\Omega^{id}\right)$.

**Algorithm 4:** 
$\mathrm{TagGen}(PK_{A},SK_{A},PK_{B},U)$

**    Ensure:** 


$\left(U,\Omega^{id}\right)$

      1:Divide the file U into *l* blocks, each forming an n×n matrix over $Z_{q}$ (padding with zeros if needed), i.e., $U:=(U_{1},\cdots ,U_{l})$, where $U_{i}\in Z_{q}^{n\times n}$ for i∈[l]      2:$u_{i}^{id}\in Z^{2m\times m_{1}}\leftarrow \mathrm{SampleLeft}\left(A_{id},A_{2}+B_{id},T_{A_{id}},D_{i}+U_{i}G,s_{3}\right)$ such that $\left[A_{id}|A_{2}+B_{id}\right]\cdot u_{i}^{id}=D_{i}+U_{i}G$, where $s_{3}\geq ∥{\tilde{T}}_{A_{id}}∥\cdot \omega \left(\sqrt{\log2m}\right)$      3:Let $\Omega^{id}={\left\{\left(U_{i},u_{i}^{id}\right)\right\}}_{i\in [l]}=\left\{\left(U_{1},u_{1}^{id}\right),\left(U_{2},u_{2}^{id}\right),\cdots ,\left(U_{l},u_{l}^{id}\right)\right\}$ as the block-tag pairs collection      4:**Return** $\left(U,\Omega^{id}\right)$

5. $\mathrm{GenProof}(PK_{A},PK_{B},\mathrm{PRF},\mathrm{PRP},\Omega^{id},CH)$ is shown in Algorithm 5. Upon inputting public keys $PK_{A},PK_{B}$, a pseudo-random function PRF, a pseudo-random permutation PRP, a block–tag pairs collection $\Omega^{id}$, and a query $CH=(|K|,\varphi_{1},\varphi_{2})$ submitted by Bob—where |K|∈[l] represents the queried data blocks’ number, and $\varphi_{1},\varphi_{2}\in Z_{q}^{*}$—the CSP outputs $\Sigma^{id}=\left\{\mu^{id},E^{id},Q^{id}\right\}$.

**Algorithm 5:** 
$\mathrm{GenProof}(PK_{A},PK_{B},\mathrm{PRF},\mathrm{PRP},\Omega^{id},CH)$

**    Ensure:** 


$\Sigma^{id}$

      1:Let K={1,2,⋯,|K|} be a set, where |K| represents the queried data blocks’ number      2:Compute $W={\mathrm{PRF}}_{\varphi_{1}}\left(K\right)$, where $W=\{W_{1},W_{2},\cdots ,W_{\left|K\right|}\}$ and $W_{j}\in Z_{q}$ for j∈[|K|]      3:Compute $V={\mathrm{PRP}}_{\varphi_{2}}\left(K\right)$, where $V=\{V_{1},V_{2},\cdots ,V_{\left|K\right|}\}$ and $V_{j}\in K$ for j∈[|K|]      4:Compute $\sigma^{id}=\sum_{j=1}^{\left|K\right|}u_{V_{j}}^{id}L_{W_{j}}$ and $\mu^{id}=\sum_{j=1}^{\left|K\right|}W_{j}U_{V_{j}}$, where ${\mathrm{GL}}_{W_{j}}=W_{j}G$. Note that we have $L_{W_{j}}=G^{-1}\left(W_{j}G\right)\in {\left\{0,1\right\}}^{m_{1}\times m_{1}}$      5:Randomly select a matrix $R^{id}\in Z_{q}^{n\times n}$ and an error matrix $S^{id}\in Z_{q}^{n\times m}$      6:Compute $Y^{id}=H_{3}\left(\mu^{id},R^{id}\right)\in Z_{q}^{2m\times m_{1}}$      7:Compute $E^{id}=\sigma^{id}+Y^{id}\in Z_{q}^{2m\times m_{1}}$      8:Compute $Q^{id}=B_{id}^{T}R^{id}+{S^{id}}^{T}\in Z_{q}^{m\times n}$      9:**Return** $\Sigma^{id}=\left\{\mu^{id},E^{id},Q^{id}\right\}$

6. $\mathrm{CheckProof}(PK_{A},PK_{B},SK_{B},CH,\Sigma^{id})$: Upon inputting public keys $PK_{A},PK_{B}$, secret key $SK_{B}$, a query $CH=(|K|,\varphi_{1},\varphi_{2})$, and a response $\Sigma^{id}$ from the CSP, Bob performs Algorithm 6.

**Algorithm 6:** 
$\mathrm{CheckProof}(PK_{A},PK_{B},SK_{B},CH,\Sigma^{id})$

**    Ensure:** 
1 (accept) or 0 (reject)      1:Compute $T_{B_{id}}^{T}Q^{id}=T_{B_{id}}^{T}\left(B_{id}^{T}R^{id}+{S^{id}}^{T}\right)=T_{B_{id}}^{T}{S^{id}}^{T}\mathrm{mod}q$      2:Compute ${S^{id}}^{T}={\left(T_{B_{id}}^{-1}\right)}^{T}T_{B_{id}}^{T}Q^{id}$, then obtain $R^{id}$ from $Q^{id}$ and $S^{id}$      3:Compute $\sigma^{id}=E^{id}-H_{3}\left(\mu^{id},R^{id}\right)\in Z_{q}^{2m\times m_{1}}$      4:Compute $W=\left\{W_{1},\cdots ,W_{\left|K\right|}\right\}={\mathrm{PRF}}_{\varphi_{1}}\left(K\right)$      5:Compute $V=\left\{V_{1},\cdots ,V_{\left|K\right|}\right\}={\mathrm{PRP}}_{\varphi_{2}}\left(K\right)$      6:Compute $D_{CH}=\sum_{j=1}^{\left|K\right|}D_{V_{j}}L_{W_{j}}$, where $L_{W_{j}}=G^{-1}\left(W_{j}G\right)\in {\left\{0,1\right\}}^{m_{1}\times m_{1}}$      7:**Return** 1 (accept) if the verification equation $\left[A_{id}|A_{2}+B_{id}\right]\cdot \sigma^{id}=D_{CH}+\mu^{id}G$ holds and $∥\sigma^{id}∥\leq s_{3}\sqrt{2mm_{1}}$, otherwise **return** 0 (reject)


### 4.2. Correctness

The correctness of our PDP scheme holds if all parties—the data owner, CSP, and designated verifier—follow the prescribed construction. Specifically, the CSP generates the PDP proof using **GenProof**, and the designated verifier checks it with **CheckProof**.

**Lemma** **8** (Correctness)**.** *If the parties act honestly, the challenge–response will pass verification, ensuring that the proposed scheme meets correctness.*

**Proof.**  For each j∈[|K|], $W_{j}\in Z_{q}$, and $V_{j}\in K$, we obtain

(3) $$A\sigma_{V_{j}}^{id}=D_{V_{j}}+M_{V_{j}}G,$$

and

(4) $$A\sigma_{V_{j}}^{id}L_{W_{j}}=A\sigma_{V_{j}}^{id}G^{-1}\left(W_{j}G\right),$$

where $A=\left[A_{id}|A_{2}+B_{id}\right]$.

Then, we have

(5) $$A\sigma_{V_{j}}^{id}L_{W_{j}}=D_{V_{j}}L_{W_{j}}+M_{V_{j}}GG^{-1}\left(W_{j}G\right)=D_{V_{j}}L_{W_{j}}+W_{j}M_{V_{j}}G.$$

For all j∈[|K|], we obtain

(6) $$\sum_{j=1}^{\left|K\right|}A\sigma_{V_{j}}^{id}L_{W_{j}}=\sum_{j=1}^{\left|K\right|}D_{V_{j}}L_{W_{j}}+\sum_{j=1}^{\left|K\right|}W_{j}M_{V_{j}}G=D_{CH}+\mu_{id}G,$$

where $D_{CH}=\sum_{j=1}^{\left|K\right|}D_{V_{j}}L_{W_{j}}$.

Hence, the verification equation $\left[A_{id}|A_{2}+B_{id}\right]\cdot \sigma^{id}=D_{CH}+\mu^{id}G$ holds. □

## 5. Security Analysis

### 5.1. Unforgeability

Assume that there exists an adversary A, as defined in Section 3.3; we design an algorithm B that utilizes a forged but valid PDP proof provided by A to solve the ${\mathrm{SIS}}_{n,m_{0},q,\beta }$ problem. Let $U^{\prime }:=\left\{U_{1}^{\prime },\cdots ,U_{\gamma }^{\prime }\right\}$ be the set (possibly empty) under the target identities $id_{A^{*}}$ and $id_{B^{*}}$, where A will generate a forgery with 0≤γ≤l.

**Theorem** **1** (Unforgeability)**.** *If a malicious CSP (adversary) succeeds in the security game with overwhelming probability ${\mathrm{Adv}}_{A}$, then the ${\mathrm{SIS}}_{n,m_{0},q,\beta }$ problem can also be solved with at least the same probability. Given the assumed hardness of the ${\mathrm{SIS}}_{n,m_{0},q,\beta }$ problem, the proposed scheme achieves selective unforgeability.*

**Proof.** 

- **Invocation:** B is given a ${\mathrm{SIS}}_{n,m_{0},q,\beta }$ assumption’s random instance, i.e., $A_{0}\in Z_{q}^{n\times m_{0}}$, and is requested to return a solution $e\in Z^{m_{0}}\setminus \left\{0\right\}$ with ||e||≤β such that $A_{0}e=0\mathrm{mod}q$.
- **Initial:** A announces to C the target identities $id_{A^{*}}$ and $id_{B^{*}}$.
- **Setup:** B employs the algorithm outlined below, utilizing $A_{0}$ from the SIS challenge as follows:
  1. Set $n,m_{0},m_{1},q$, $m_{1}=n\left\lceil \log q\right\rceil$ and $m=m_{0}+m_{1}$.
  2. Let $s_{1},s_{2},s_{3}$ denote the Gaussian parameters.
  3. $\left(B,T_{B}\right)\leftarrow \mathrm{TrapGen}\left(1^{n},1^{m_{1}},q\right)$.
  4. Randomly pick the matrices $M_{1}$ from ${\left\{-1,1\right\}}^{m_{0}\times m_{1}}$, $M_{2}$ from ${\left\{-1,1\right\}}^{m_{0}\times m}$, and $M_{3}\in D_{m\times m}$.
  5. Set $A_{1}=A_{0}M_{1}-H_{1}\left(id_{A^{*}}\right)B$ and $A_{2}=A_{0}M_{2}-\left[A_{0}|A_{0}M_{1}\right]M_{3}^{-1}$.
  6. Randomly sample ${\left[u_{i,1}^{id_{A^{*}}T}|u_{i,2}^{id_{A^{*}}T}\right]}^{T}\leftarrow {\left(D_{Z^{m},s_{3}}\right)}^{2m}$ for i∈[γ] as “pre-signatures”, and then set
    (7) $$D_{i}=A_{{id}_{A^{*}}}\left(\begin{array}{c} u_{i,1}^{id_{A^{*}}}\\ u_{i,2}^{id_{A^{*}}} \end{array}\right)-U_{i}^{\prime }G,$$
    where $A_{{id}_{A^{*}}}=\left[\left(A_{0}|A_{0}M_{1}\right)|A_{0}M_{2}\right]=A_{0}\left[\left(I_{m_{0}}|M_{1}\right)|M_{2}\right]$.
  7. Randomly pick l−γ matrices ${\left\{D_{j}\right\}}_{j\in [[l]\setminus [\gamma ]]}\in Z_{q}^{m\times m_{1}}$ for j∈[l]∖[γ].
  8. Output the public parameter $params=\left(A_{0},A_{1},A_{2},B,{\left\{D_{i}\right\}}_{i\in \left[l\right]}\right)$.
  The inquiry process of B simulation random oracle $H_{2}$ and $H_{3}$ is as follows. B initializes two empty lists $L_{1}$ and $L_{2}$. The following outlines the queries and steps:
- **$H_{2}$ queries.** Upon inputting an identity $id_{B}$, perform the following:
  1. If $id_{B}\neq id_{B^{*}}$, there exists $H_{2}\left(id_{B}\right)$ in list $L_{1}$; return $H_{2}\left(id_{B}\right)=F_{id_{B}}$ to A. Otherwise, run the algorithm $\mathrm{SampleRwithBasis}\left(A_{id}\right)$ to generate $F_{id_{B}}\in D_{m\times m},B_{id}=A_{id}F_{id_{B}}^{-1}\in Z_{q}^{n\times m}$ and $\Lambda_{q}^{⊥}\left(B_{id}\right)$’s basis $T_{B_{id}}$, where $A_{id}=\left[A_{0}|A_{0}M_{1}\right]$. Then, store $\left(id_{B},F_{id_{B}},B_{id},T_{B_{id}}\right)$ into list $L_{1}$ and return $H_{2}\left(id_{B}\right)=F_{id_{B}}$ to A.
  2. If $id_{B}=id_{B^{*}}$, then add $\left(id_{B},M_{3},A_{id}M_{3}^{-1},⊥\right)$ into list $L_{1}$ and return $H_{2}\left(id_{B^{*}}\right)=M_{3}$.
- **Key Queries.** Upon inputting identities $id_{A},id_{B}$ (except $id_{A^{*}},id_{B^{*}}$), perform the following:
  1. $T_{A_{id}}\leftarrow \mathrm{SampleBasisRight}\left(A_{0},\left(H_{1}\left(id_{A}\right)-H_{1}\left(id_{A^{*}}\right)\right)B,M_{1},T_{B},s_{1}\right)$.
  2. Search for $id_{B}$ in $L_{1}$ and return the associated $T_{B}$.
  3. Output $\left(PK_{A},SK_{A}\right)=\left(A_{id},T_{A_{id}}\right)$ and $\left(PK_{B},SK_{B}\right)=\left(B_{id},T_{B_{id}}\right)$.
- **Block–tag pairs queries.** Upon receiving the set $U^{\prime }$ from A, B responds with block–tag pairs ${\left\{\left(U_{i}^{\prime },u_{i}^{\prime id_{A^{*}}}\right)\right\}}_{i\in [\gamma ]}$, where ${\left\{u_{i}^{\prime id_{A^{*}}}\right\}}_{i\in [\gamma ]}={\left\{{\left[u_{i,1}^{id_{A^{*}}T}|u_{i,2}^{id_{A^{*}}T}\right]}^{T}\right\}}_{i\in [\gamma ]}$ are precomputed during **Setup** phase.
- **$H_{3}$ queries.** Upon inputting $\mu^{id}$, perform the following:
  1. If there exists $\mu^{id}$ in list $L_{2}$, return the corresponding value $Y^{id}$ and $Q^{id}$ to A.
  2. Otherwise, B randomly picks a matrix $R^{id}\in Z_{q}^{n\times n}$ and an error matrix $S^{id}\in Z_{q}^{n\times m}$, computes
    (8) $$Y^{id}=H_{3}\left(\mu^{id},R^{id}\right),$$
    and
    (9) $$Q^{id}=B_{id}^{T}R^{id}+{S^{id}}^{T},$$
    and then adds $\left(\mu^{id},Y^{id},Q^{id}\right)$ into $L_{2}$ and returns $Y^{id}$ and $Q^{id}$.
- **PDP proof queries.** A issues a challenge $CH=(|K|,\varphi_{1},\varphi_{2})$, limited to the set $U^{\prime }$, where |K|∈[γ] and $\varphi_{1},\varphi_{2}\in Z_{q}^{*}$. The challenger uses ${\left\{\left(U_{i}^{\prime },u_{i}^{id_{A^{*}}}\right)\right\}}_{i\in [\gamma ]}$ to produce a valid PDP proof $\Sigma^{{id}_{A^{*}}}=\left\{\mu^{{id}_{A^{*}}},E^{{id}_{A^{*}}},Q^{{id}_{A^{*}}}\right\}$ by running Algorithm 5 and transmits it to A.
- **Challenge.** B generates a challenge $CH^{*}=(|K^{*}|,\varphi_{1}^{*},\varphi_{2}^{*})$ restricted to $U^{\prime }$, where $0<|K^{*}|\leq \gamma$ and $\varphi_{1}^{*},\varphi_{2}^{*}\in Z_{q}^{*}$, and transmits it to A.
- **Forgery.** A returns a forgery ${\hat{\Sigma }}^{{id}_{A^{*}}}=\left\{{\hat{\mu }}^{{id}_{A^{*}}},{\hat{E}}^{{id}_{A^{*}}},{\hat{Q}}^{{id}_{A^{*}}}\right\}$. If ${\hat{\Sigma }}^{{id}_{A^{*}}}$ passes the verifier’s check with overwhelming probability ${\mathrm{Adv}}_{A}$, then the Equation (10) holds, with ${\mathrm{Adv}}_{A}$, as
  (10) $$A_{id_{A^{*}}}{\hat{\sigma }}^{id_{A^{*}}}=D_{CH}+{\hat{\mu }}^{{id}_{A^{*}}}G,$$
  where $A_{{id}_{A^{*}}}=\left[\left(A_{0}|A_{0}M_{1}\right)|A_{0}M_{2}\right]=A_{0}\left[\left(I_{m_{0}}|M_{1}\right)|M_{2}\right]$ and $D_{CH}=\sum_{j=1}^{|K^{*}|}D_{V_{j}}L_{W_{j}}$.
  Let $\Sigma^{{id}_{A^{*}}}=\left\{\mu^{{id}_{A^{*}}},E^{{id}_{A^{*}}},Q^{{id}_{A^{*}}}\right\}$ be computed honestly. Here,
  (11) $$\mu^{{id}_{A^{*}}}=\sum_{j=1}^{|K^{*}|}W_{j}U_{V_{j}}^{\prime },$$
  (12) $$E^{{id}_{A^{*}}}=\sigma^{{id}_{A^{*}}}+H_{3}\left(\mu^{{id}_{A^{*}}},R^{{id}_{A^{*}}}\right),$$
  and
  (13) $$Q^{{id}_{A^{*}}}=B_{{id}_{A^{*}}}^{T}R^{{id}_{A^{*}}}+{S^{{id}_{A^{*}}}}^{T},$$
  where $W=\left\{W_{1},\cdots ,W_{|K^{*}|}\right\}={\mathrm{PRF}}_{\varphi_{1}^{*}}\left(K^{*}\right)$, and $V=\left\{V_{1},\cdots ,V_{|K^{*}|}\right\}={\mathrm{PRP}}_{\varphi_{2}^{*}}\left(K^{*}\right)$. Thus, we obtain
  (14) $$A_{id_{A^{*}}}\sigma^{id_{A^{*}}}=D_{CH}+\mu^{{id}_{A^{*}}}G.$$
  Hence, we have
  (15) $$A_{id_{A^{*}}}\left({\hat{\sigma }}^{id_{A^{*}}}-\sigma^{id_{A^{*}}}\right)=\left({\hat{\mu }}^{id_{A^{*}}}-\mu^{id_{A^{*}}}\right)G,$$
  which holds with overwhelming probability ${\mathrm{Adv}}_{A}$.
  Next, we show that if adversary A succeeds in the security game with non-negligible advantage ${\mathrm{Adv}}_{A}$, then challenger B can solve an instance of the ${\mathrm{SIS}}_{n,m_{0},q,\beta }$ problem with at least the same probability.
  Let ${\hat{\sigma }}^{{\mathrm{id}}_{A^{*}}}={\left[{\hat{u}}_{1}^{{\mathrm{id}}_{A^{*}}T}|{\hat{u}}_{2}^{{\mathrm{id}}_{A^{*}}T}\right]}^{T}$ be the forged response produced by A. Since ${\hat{\Sigma }}^{{\mathrm{id}}_{A^{*}}}\neq \Sigma^{{\mathrm{id}}_{A^{*}}}$, at least one of the following must hold: (i) ${\hat{\mu }}^{{\mathrm{id}}_{A^{*}}}=\mu^{{\mathrm{id}}_{A^{*}}}$, where ${\hat{\sigma }}^{{\mathrm{id}}_{A^{*}}}\neq \sigma^{{\mathrm{id}}_{A^{*}}}$; (ii) ${\hat{\mu }}^{{\mathrm{id}}_{A^{*}}}\neq \mu^{{\mathrm{id}}_{A^{*}}},$ where ${\hat{\sigma }}^{{\mathrm{id}}_{A^{*}}}=\sigma^{{\mathrm{id}}_{A^{*}}}$; (iii) ${\hat{\mu }}^{{\mathrm{id}}_{A^{*}}}\neq \mu^{{\mathrm{id}}_{A^{*}}},$ where ${\hat{\sigma }}^{{\mathrm{id}}_{A^{*}}}\neq \sigma^{{\mathrm{id}}_{A^{*}}}$.
  We analyze the above cases:
  – **Case 1:** If the forged tag differs while the file block remains unchanged, then B outputs $\left[\left[I_{m_{0}}|M_{1}\right]{\left[{\hat{u}}_{1}^{id_{A^{*}}T}-u_{1}^{id_{A^{*}}T}\right]}^{T}+M_{2}{\left[{\hat{u}}_{2}^{id_{A^{*}}T}-u_{2}^{id_{A^{*}}T}\right]}^{T}\right]$ as the ${\mathrm{SIS}}_{n,m_{0},q,\beta }$ solution.
  – **Case 2:** If the file block differ but the tags are identical, then this contradicts Equation (15). Thus, this case is not possible.
  – **Case 3:** If both the file block and tag differ, then B outputs $\left[\left[I_{m_{0}}|M_{1}\right]{\left[{\hat{u}}_{1}^{id_{A^{*}}T}-u_{1}^{id_{A^{*}}T}\right]}^{T}+M_{2}{\left[{\hat{u}}_{2}^{id_{A^{*}}T}-u_{2}^{id_{A^{*}}T}\right]}^{T}\right]T_{G}$ as the ${\mathrm{SIS}}_{n,m_{0},q,\beta }$ solution, where $T_{G}$ is a short basis of $\Lambda_{q}\left(G\right)$.

□

### 5.2. Indistinguishability

In addition to unforgeability, the proposed scheme also satisfies indistinguishability. This property simply means that the simulated experiment and the real algorithm are indistinguishable from the adversary’s perspective.

**Theorem** **2** (Indistinguishability)**.** Let $\left\{params,PK_{A},SK_{A},PK_{B},SK_{B},\Sigma^{id}\right\}$ and $\{params^{*},PK_{A}^{*},$$SK_{A}^{*},PK_{B}^{*},SK_{B}^{*},\Sigma^{{id}^{*}}\}$ be the outputs of the **Setup**, **KeyGenS**, **KeyGenV**, **TagGen**, and **GenProof** algorithms in real and simulated execution, respectively. We demonstrate that the two distributions are statistically indistinguishable.

**Proof.**  The differences are as follows in the execution of the algorithm:

- **Setup:** In the real Setup algorithm, $A_{0}\in Z_{q}^{n\times m_{0}}$ and its trapdoor $T_{A_{0}}$ are obtained by using the TrapGen algorithm, and matrices $\left(A_{1},A_{2},B,{\left\{D_{i}\right\}}_{i\in [l]}\right)$ are selected uniformly at random. In the simulated Setup algorithm, the SIS generator uniformly randomly selects $A_{0}$ without its trapdoor. Define $A_{1}=A_{0}M_{1}-H\left(id_{A^{*}}\right)B$ and $A_{2}=A_{0}M_{2}-\left[A_{0}|A_{0}M_{1}\right]M_{3}^{-1}$, where $M_{1}\in {\left\{-1,1\right\}}^{m_{0}\times m_{1}}$, $M_{2}\in {\left\{-1,1\right\}}^{m_{0}\times m}$ and $M_{3}\in D_{m\times m}$ are selected uniformly randomly. B is sampled using the Trapdoor algorithm. Additionally, set $D_{i}=\left[\left(A_{0}|A_{0}M_{1}\right)|A_{0}M_{2}\right]\left[\begin{array}{c} u_{i,1}^{id}\\ u_{i,2}^{id} \end{array}\right]-U_{i}G$ for i∈[γ], where ${\left[\begin{array}{c} {u_{i,1}^{id}}^{T}|{u_{i,2}^{id}}^{T} \end{array}\right]}^{T}\leftarrow {\left(D_{Z^{m},s_{3}}\right)}^{2m}$, and for j∈[l]∖[γ], sample uniform random $D_{j}\in Z_{q}^{n\times m_{1}}$.
- **KeyGenS:** In real KeyGenS algorithm, the trapdoor $T_{A_{id}}$ of $A_{id}$ is generated by running SampleBasisLeft. In the simulated KeyGenS algorithm, $T_{A_{id}}$ is produced using SampleBasisRight.
- **KeyGenV:** In real KeyGenV algorithm, the trapdoor $T_{B_{id}}$ of $B_{id}$ is produced using NewBasisDel. In the simulated KeyGen algorithm, $T_{B_{id}}$ is generated using SampleRwithBasis.
- **TagGen:** In the real TagGen algorithm, $u_{i}^{id}$ is generated using the SampleLeft algorithm where i∈[l]. In the simulated TagGen algorithm, $u_{i}^{id}$ is pre-produced during the **Setup** phase.
- **GenProof:** In the real GenProof algorithm, $R^{id}\in Z_{q}^{n\times n}$ and $S^{id}\in Z_{q}^{n\times m}$ are uniformly selected randomly. $u_{i}^{id}$ generated by the SampleLeft algorithm is used to produce $\sigma^{id}$, where i∈[l]. $\Sigma^{id}$ is then derived from $R^{id},S^{id}$ and $u_{i}^{id}$. In the simulated GenProof algorithm, the matrix $R^{id}=\left[A_{0}|A_{0}M_{1}\right]M_{4}$ for $M_{4}\in {\left\{-1,1\right\}}^{m\times n}$, the matrix $S^{id}={\left(\left[A_{0}|A_{0}M_{1}\right]M_{5}\right)}^{T}$ for $M_{5}\in {\left\{-1,1\right\}}^{m\times m}$, and $u_{i}^{id}$ are pre-produced during the **Setup** phase, and $\Sigma^{id}$ is obtained by using $R^{id},S^{id}$ and $u_{i}^{id}$.

Now, we argue that the distribution $\left(A_{0},A_{1},A_{2},B,{\left\{D_{i}\right\}}_{i\in [l]}\right)$ is statistically indistinguishable in both real and simulated executions. By Lemma 1, it has $(A_{0},[A_{1}|A_{2}|D_{1}|D_{2}\left|\cdots \right|$$D_{l})\approx (A_{0},[A_{0}M_{1}-H\left(id_{A}\right)B|A_{0}M_{2}-\left[A_{0}|A_{0}M_{1}\right]M_{3}^{-1}|D_{1}|D_{2}\left|\cdots \right|D_{l}])$, where $D_{i}=\left[A_{0}|A_{0}M_{1}\right]u_{i,1}^{id}+A_{0}M_{2}u_{i,2}^{id}-U_{i}G$ for i∈[γ], and $D_{j}\in Z_{q}^{n\times m_{1}}$ for j∈[l]∖[γ]. Here, ${\left[\begin{array}{c} {u_{i,1}^{id}}^{T}|{u_{i,2}^{id}}^{T} \end{array}\right]}^{T}\leftarrow {\left(D_{Z^{m},s_{3}}\right)}^{2m}$. In both executions, $\left(B,T_{B}\right)$ is produced identically. Hence, the distribution of $\left(A_{0},A_{1},A_{2},B,{\left\{D_{i}\right\}}_{i\in [l]}\right)$ is statistically indistinguishable in both cases.

For a key query on an identity $id_{A}$, the algorithms **SampleBasisLeft** and **SampleBasisRight** are invoked to generate the corresponding trapdoor. By leveraging Lemmas 3 and 4, we ensure that the output of these algorithms is statistically close to a sample from the discrete Gaussian distribution $D_{\Lambda_{\left(A_{id}\right),s_{1}}}$, assuming that the Gaussian parameter $s_{1}$ is chosen to be sufficiently large. This implies that the generated trapdoor is statistically independent of any specific structure or information leakage. Similarly, for another identity $id_{B}$, the **NewBasisDel** and **SampleRwithBasis** algorithms are used to derive the trapdoor. According to Lemmas 5 and 6, their outputs are computationally and statistically indistinguishable, ensuring that no adversary can distinguish whether the trapdoor was generated in the real execution or through simulation. Thus, in both executions, $T_{A_{id}}\left(T_{B_{id}}\right)$ is statistically indistinguishable.

According to Lemmas 3 and 4, the distributions of public parameters, public/secret keys for identities, and tags associated with challenge files are statistically indistinguishable in both the real and simulated executions. Therefore, an adversary cannot leverage these components to distinguish between the two settings.

For the **GenProof** algorithm in both executions, considering the secure hash function $H_{3}$ and the LWE instance, the $\Sigma^{id}=\left\{\mu^{id},E^{id},Q^{id}\right\}$ values produced by the two executions are statistically close to uniform and random. □

### 5.3. Robustness

**Theorem** **3** (Robustness)**.** *Our scheme meets robustness if the LWE problem is hard.*

**Proof.**  We define robustness as the guarantee that only the designated verifier, authorized by the data owner, is capable of successfully validating the integrity of the data.

In the proposed scheme, the verifier-specific component of the proof response is defined as $Q^{id}=B_{id}^{T}\cdot R^{id}+S^{id\;T}$, which is an LWE instance based on the robustness assumption of the LWE problem.

Under the LWE assumption, the distribution of $Q^{id}$ is indistinguishable from a uniformly random matrix over $Z_{q}^{n\times m}$. Therefore, without knowledge of the designated verifier’s private key, it is infeasible for any adversary—including other verifiers, CSPs, or external attackers—to recover the underlying secret $R^{id}$.

If such an adversary A could recover $R^{id}$ from $Q^{id}$ without being the designated verifier, this would imply that A can distinguish $Q^{id}$ from random and invert the LWE function, thereby solving an instance of the LWE problem—contradicting our hardness assumption.

Therefore, under the assumption that LWE is hard, our scheme ensures that only the designated verifier can perform valid proof verification, satisfying the definition of robustness. □

### 5.4. Parameter Selection

Since the proposed PDP scheme is based on the trapdoor construction [33] and utilizes a specific leveled IB-FHS for signature generation, the parameter relationships were analyzed with these factors in mind. Following the computational formulas provided in [32,34], we derived the parameters of the scheme accordingly. The parameters are presented in Table 2, ensuring that the scheme achieved the specified level of security.

Although the SIS and LWE are widely considered hard problems for post-quantum cryptography, their security may be affected by unforeseen advances in quantum algorithms or future quantum computing capabilities. In such cases, it would be necessary to increase the lattice dimension *n* and the modulus *q* to maintain an adequate security level.

## 6. Performance Evaluation

### 6.1. Functionality Comparison

As presented in Table 3, several existing schemes, like Deng et al.’s (2023) [10], Wu et al.’s [21], and Yan et al.’s [27], do not incorporate an identity-based cryptosystem (IBC), thereby limiting their applicability in environments that demand streamlined identity management. Although Deng et al.’s (2024) [11], Luo et al.’s [14], and Zhou et al.’s [35] schemes support an IBC, they lack the capability for designated third-party auditing, which is crucial in applications such as cloud auditing—especially in scenarios where it is necessary to guard against potential malicious auditors tampering with or forging audit results.

With respect to security assumptions, certain schemes (e.g., [10,11,21,27]) rely on traditional number-theoretic problems like DL and CDH problems, which are potentially vulnerable in the presence of quantum adversaries. While schemes such as Luo et al.’s [14], Sasikala and Bindu’s [19], and Zhou et al.’s [35] adopt lattice-based hardness assumptions (e.g., SIS) and offer a degree of quantum resistance, they still fail to provide a complete set of functionalities, as none of them simultaneously support both IBC and feature a designated verifier.

In contrast, the PDP scheme proposed in this work offers a more comprehensive and well-balanced design. It supports both IBC and designated third-party validation, thereby enhancing its practical deployability and ensuring stronger accountability. Moreover, this scheme relies on the hardness of both the SIS and LWE problems, offering strong resistance against quantum attacks. Consequently, the proposed scheme theoretically exhibits strong resistance against quantum attacks while fulfilling advanced functional requirements, making it particularly suitable for practical deployment and applications.

### 6.2. Time and Space Complexity

Table 4 presents a comparison between our scheme and the scheme by Luo et al. [14] in terms of time and space complexity across three core phases: **TagGen**, **GenProof**, and **CheckProof**. This comparison aims to quantitatively evaluate the computational efficiency and resource consumption of different designs.

In the **TagGen** phase, the time complexity of our scheme is $O(lnm_{1}^{2})$, and the space complexity is $O(l\left(n^{2}+nm_{1}\right))$. By contrast, the scheme by Luo et al. [14] exhibits a time complexity of $O(l\left(n^{2}m_{1}+\left(m+m_{1}\right)m_{1}\right))$ and a space complexity of $O(l\left(n^{2}+\left(m+m_{1}\right)m_{1}\right))$. Our scheme incurs lower computational and storage overhead in the **TagGen** phase, making it especially suitable for efficient large-scale data tag generation.

Moreover, in the **GenProof** and **CheckProof** phases, our scheme incorporates a designated verifier mechanism, which embeds verifier identity information into authentication tags and proofs, thereby enabling control over verification rights. This mechanism ensures that only authorized verifiers can perform verification, significantly enhancing the system’s support for controlled auditability.

Although this mechanism introduces additional structure and computation—leading to slightly higher complexity in the **GenProof** and **CheckProof** phases compared to the Luo et al. [14] scheme—the overall complexity remains within a practical polynomial range. The overhead remains moderate under typical parameters. In summary, our scheme achieves a well-balanced tradeoff between enhanced security capabilities and acceptable computational cost, offering both flexibility and efficiency.

### 6.3. Communication and Storage Cost

As shown in Table 5, we evaluated the communication and storage overhead of our scheme in comparison with several lattice-based PDP constructions, focusing on public and secret key (PK + SK) size, tag size, and proof size. The findings indicate that our scheme achieves a balanced tradeoff between efficiency and functionality.

In terms of key size, our scheme requires $(nm+m^{2})\log q$ bits, which matches the construction proposed by Sasikala and Bindu [19] and is smaller than those by Luo et al. [14] and Zhou et al. [35], demonstrating efficient storage without compromising functionality. Regarding tag size, our design incurs $2mm_{1}\log q$, which is slightly larger than that in Sasikala and Bindu’s [19] work and the approach by Zhou et al. [35], but remains considerably smaller than the quadratic growth in Luo et al.’s scheme [14], striking a balance between efficiency and security. With respect to proof size, our scheme requires $(n^{2}+\left(n+2m_{1}\right)m)\log q$ bits, which is larger than the proof sizes in Sasikala and Bindu’s [19] and Zhou et al.’s [35] designs, but still more efficient than the construction proposed by Luo et al. [14]. Notably, the increased proof size in our design stems primarily from the incorporation of a designated verifier mechanism. This feature enhances privacy and accountability in audit scenarios, thus slightly increasing the communication cost during the proof generation phase.

In summary, the proposed scheme maintains reasonable efficiency while supporting IBS, designated third-party validation, and post-quantum security. It demonstrates strong practicality and is particularly well suited for secure cloud storage applications in the post-quantum era.

### 6.4. Computation Cost

In this section, we present the experiments conducted to evaluate the proposed PDP scheme, which were executed using MATLAB 2020b on a system with an Intel(R) Core(TM) i5-13500H processor (2.60 GHz) and 16GB of RAM. The file size *M* used in the experiments was approximately 1MB.

The computation cost for the proposed PDP scheme algorithms (**TagGen**, **GenProof**, and **CheckProof**) was assessed for different lattice dimensions—denoted by *n*. For a file of size M≈1 MB, when n=128, the corresponding number of data blocks came out to 21, and when n=256, the number of data blocks came out to 5. The designated verifier can challenge all the data blocks to achieve a detection probability of 100%.

**Figure 3:**
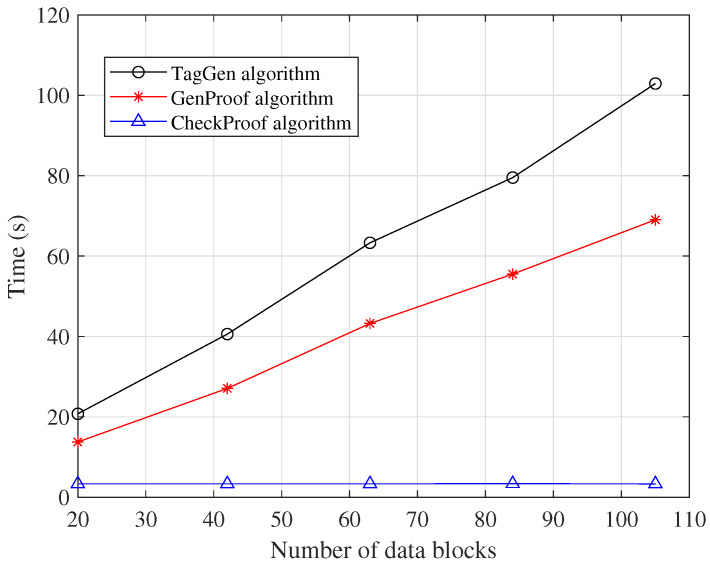
Computation time (s).

The results of the computation time for n=128 and n=256 are shown in Table 6. Figure 3 illustrates the computation time of the **TagGen**, **GenProof**, and **CheckProof** algorithms with respect to the number of data blocks. As shown in the figure, both the **TagGen** and **GenProof** algorithms exhibited a linear increase in computation time as the number of data blocks grew. Notably, in the **CheckProof** algorithm, the verifier could compute the matrix $D_{CH}$ offline, which helped reduce the verification time. As a result, the computation time for **CheckProof** remained relatively stable even as the number of data blocks increased.

Compared with existing PDP schemes based on various insecure number-theoretic assumptions, the proposed PDP scheme requires larger parameters, including public and private keys, and exhibits higher computational costs. However, it offers promising potential for post-quantum cryptography. Compared with existing lattice-based PDP schemes, the additional overhead primarily stems from the introduction of the designated verifier mechanism. Moreover, in certain practical applications and implementations, the proposed scheme inherits the same limitations as the leveled FHS scheme—namely, suboptimal performance.

## 7. Conclusions

This paper presents a lattice-based PDP scheme that incorporates a designated verifier using a leveled IB-FHS. The scheme was proven secure under SIS and LWE assumptions in the random oracle model, confirming its theoretical soundness and feasibility. We have also evaluated the effectiveness and practicality of the proposed scheme through performance comparisons with existing PDP schemes in terms of computation, storage, and communication cost. The results demonstrate that our approach achieves a favorable balance between security and efficiency, making it well suited for authorization-based cloud storage auditing scenarios.

However, we also acknowledge several limitations that merit further exploration. First, compared to traditional PDP schemes, the increased computational overhead of the proposed construction is primarily attributable to its reliance on lattice-based cryptography and the inclusion of a designated verifier mechanism. Second, the current design is centered around a designated verifier and does not consider the complexities introduced by multi-tenant cloud environments, where ensuring data isolation and enforcing fine-grained access control among tenants are essential. Third, although the scheme includes theoretical performance estimates, real-world deployment may involve additional overhead and integration challenges that require practical validation. Furthermore, transitioning the construction to the standard model remains an important and meaningful direction for enhancing its theoretical robustness. As future work, we plan to (1) optimize the efficiency of the core algorithms to reduce computational costs; (2) extend the proposed scheme to support secure auditing in multi-tenant cloud settings with proper isolation mechanisms and delegated verification control; and (3) develop a standard model instantiation to eliminate reliance on idealized cryptographic assumptions.

## Figures and Tables

**Table 1:** Symbol description.

Symbols	Definitions
$a\in Z_{q}$	Random numbers on integer module *q* spaces
$z\in Z_{q}^{n}$	*n*-dimensional vectors on integer module spaces *q*
$Q\in Z_{q}^{n\times m}$	*n*-row *m*-column matrices on integer module *q* space
$Q^{T}$	Transpose matrix of Q
$\tilde{Q}$	Gram–Schmidt orthogonalization of a matrix Q
∥Q∥	Matrix Q’ $l_{2}$-norm
[·|·]	Horizontal concatenation of vectors or matrices
[t]	Set {1,2,⋯,t}
O(·)	Asymptotic upper bound

**Table 2:** Parameter set for Gaussian parameter r=8.

$L_{c}$	0	0	0	1	1	5	10	15
λ	75	100	128	75	128	75	75	128
*n*	157	178	544	497	1127	1256	4624	11,912
*m*	4059	4610	26,099	26,828	94,618	143,088	1,747,591	8,814,469
logq	13	13	24	27	42	57	189	370

$L_{c}$—circuit depth; λ—a security parameter; n,m—the parameters employed in trapdoor function; *q*—a large integer.

**Table 3:** Functionality comparisons with related schemes.

	Items	IBC	Designated Verifier	Assumption	QAR
Schemes					
Deng et al. (2023) [10]		✗	✗	DL	✗
Deng et al. (2024) [11]		✓	✗	CDH	✗
Luo et al. [14]		✓	✗	SIS	✓
Sasikala and Bindu [19]		✗	✗	SIS	✓
Wu et al. [21]		✗	✓	DL	✗
Yan et al. [27]		✗	✓	CDH	✗
Zhou et al. [35]		✓	✗	SIS	✓
Our Scheme		✓	✓	SIS, LWE	✓

IBC—identity-based cryptosystem; QAR—quantum attacks resistance; DL—discrete logarithm problem; ✓ indicates that the scheme has this feature; ✗ indicates that the scheme does not have this feature.

**Table 4:** Time and space complexity comparisons with related scheme.

Schemes	Algorithms	Time Complexity	Space Complexity
Luo et al. [14]	**TagGen**	$O(l\left(n^{2}m_{1}+\left(m+m_{1}\right)m_{1}\right))$	$O(l\left(n^{2}+\left(m+m_{1}\right)m_{1}\right))$
	**GenProof**	$O(|K|\left(n^{2}+\left(m+m_{1}\right)m_{1}^{2}\right))$	$O(|K|\left(n^{2}+\left(m+m_{1}\right)m_{1}\right))$
	**CheckProof**	$O(|K|\left(\left(m+m_{1}\right)m_{1}^{2}+nm_{1}\right))$	$O(\left(m+m_{1}\right)+nm_{1}+|K|)$
Our Scheme	**TagGen**	$O(lnm_{1}^{2})$	$O(l\left(n^{2}+nm_{1}\right))$
	**GenProof**	$O(|K|n^{2}+nm)$	$O(|K|\left(n^{2}+m_{1}\right)+nm)$
	**CheckProof**	$O(|K|mm_{1}+\left(m+m_{1}\right)m_{1})$	$O(|K|+mm_{1}+n^{2})$

$m_{1}=n\left\lceil \log q\right\rceil$; *l*—the maximum number of data blocks; |K|—the number of the queried data blocks.

**Table 5:** Communication and storage comparisons with related schemes.

	Items	PK + SK Size	Tag Size	Proof Size
Schemes				
Luo et al. [14]		$(nm+nm_{1}+mm_{1})\log q$	$\left(m+m_{1}\right)m_{1}\log q$	$(n^{2}+\left(m+m_{1}\right)m_{1})\log q$
Sasikala and Bindu [19]		$(nm+m^{2})\log q$	mlogq	(n+2m)logq
Zhou et al. [35]		$(nm+m^{2}+2\bar{l}m)\log q$	mlogq	(n+2m)logq
Our Scheme		$(nm+m^{2})\log q$	$2mm_{1}\log q$	$(n^{2}+\left(n+2m_{1}\right)m)\log q$

PK—public key; SK—secret key; $m=m_{1}+m_{0}$; $m_{1}=n\left\lceil \log q\right\rceil$; $\bar{l}$—the bit length of a file block.

**Table 6:** Computation cost (s).

	Algorithms	TagGen	GenProof	CheckProof
*n*				
128		20.76	13.75	3.33
256		36.66	30.67	5.17

## Data Availability

The data that support the findings of this study are available from the corresponding author upon reasonable request.
